# The impact of surgical site infection—a cost analysis

**DOI:** 10.1007/s00423-021-02346-y

**Published:** 2021-10-14

**Authors:** Rahel M. Strobel, Marja Leonhardt, Frank Förster, Konrad Neumann, Leonard A. Lobbes, Claudia Seifarth, Lucas D. Lee, Christian H. W. Schineis, Carsten Kamphues, Benjamin Weixler, Martin E. Kreis, Johannes C. Lauscher

**Affiliations:** 1 Department of General, Visceral and Vascular Surgery, Charité Campus Benjamin Franklin, Hindenburgdamm 30, 12203 Berlin, Germany; 2 Innlandet Hospital Trust, Norwegian National Advisory Unit On Concurrent Substance Abuse and Mental Health Disorders, Brumunddal, Norway; 3 Corporate Controlling, Charité Campus Mitte, Charitéplatz 2, 10117 Berlin, Germany; 4 Institute of Biometry and Clinical Epidemiology, Charité Universitätsmedizin Berlin, Freie Universität Berlin, Humboldt-Universität Zu Berlin and Berlin Institute of Health, Charitéplatz 1, 10117 Berlin and Berlin Institute of Health (BIH), Anna-Louisa-Karsch Str. 2, 10178 Berlin, Germany

**Keywords:** Surgical site infection, Cost analysis, Visceral surgery, Laparotomy

## Abstract

**Purpose:**

Surgical site infection (SSI) occurs in up to 25% of patients after elective laparotomy. We aimed to determine the effect of SSI on healthcare costs and patients’ quality of life.

**Methods:**

In this post hoc analysis based on the RECIPE trial, we studied a 30-day postoperative outcome of SSI in a single-center, prospective randomized controlled trial comparing subcutaneous wound irrigation with 0.04% polyhexanide to 0.9% saline after elective laparotomy. Total medical costs were analyzed accurately per patient with the tool of our corporate controlling team which is based on diagnosis-related groups in Germany.

**Results:**

Between November 2015 and May 2018, 456 patients were recruited. The overall rate of SSI was 28.2%. Overall costs of inpatient treatment were higher in the group with SSI: median 16.685 €; 19.703 USD (IQR 21.638 €; 25.552 USD) vs. median 11.235 €; 13.276 USD (IQR 11.564 €; 13.656 USD); *p* < 0.001. There was a difference in surgery costs (median 6.664 €; 7.870 USD with SSI vs. median 5.040 €; 5.952 USD without SSI; *p* = 0.001) and costs on the surgical ward (median 8.404 €; 9.924 USD with SSI vs. median 4.690 €; 5.538 USD without SSI; *p* < 0.001). Patients with SSI were less satisfied with the cosmetic result (4.3% vs. 16.2%; *p* < 0.001). Overall costs for patients who were irrigated with saline were median 12.056 €; 14.237 USD vs. median 12.793 €; 15.107 USD in the polyhexanide group (*p* = 0.52).

**Conclusion:**

SSI after elective laparotomy increased hospital costs substantially. This is an additional reason why the prevention of SSI is important. Overall costs for intraoperative wound irrigation with saline were comparable with polyhexanide.

## Introduction

Surgical site infection (SSI) is a common complication of abdominal surgery. Previous studies showed SSI rates after visceral surgery as high as 20% [1, 2]. SSI results in up to one million additional days of hospitalization per year and considerable healthcare costs in Germany [3]. In the USA, attributable costs for SSI vary between $10.443 and $25.546 per infection [4, 5]. Over the years, early discharge from the hospital became one goal of medical care. SSI is a huge burden for the patient and has a relevant impact on the patient’s satisfaction with healthcare. There is a lack of studies concerning the quality of life of patients with SSI in abdominal surgery. In the RECIPE trial (**Re**du**c**t**i**on of **p**ostoperative wound infections by antis**e**ptica?) conducted in the Department of General, Visceral and Vascular Surgery of the Charité – Campus Benjamin Franklin Berlin, we could show that intraoperative subcutaneous wound irrigation with polyhexanide reduces SSI in elective laparotomies compared to wound irrigation with saline [6].

The objective of this post hoc analysis of the RECIPE trial was to determine the impact of SSI on inpatient costs and patients’ postoperative outcome. We compared the costs of patients receiving intraoperative wound irrigation with saline and polyhexanide.

## Materials and methods

### Trial oversight

Data presented in this manuscript are based on the RECIPE trial. The RECIPE trial was a single-center, randomized controlled, prospective trial with two treatment groups comparing intraoperative subcutaneous wound irrigation with 250 ml of 0.04% polyhexanide solution (Serasept2®, Serag-Wiessner, Naila, Germany) to 250 ml of 0.9% saline in elective open or laparoscopically assisted abdominal surgery. The primary outcome data — rate of SSI 30 days postoperatively — was published previously [6]. Here, we report a post hoc analysis of this prospectively collected cohort analyzing the impact of SSI on inpatient costs and 30-day postoperative outcome.

The RECIPE trial was an investigator-initiated Medicinal Products Act trial. The study protocol was approved by the State Office for Health and Social Affairs Berlin (Study Protocol code RECIPE2014; EudraCT number: 2014–001,551-22) and the Federal Institute for Drugs and Medical Devices. The trial was conducted in accordance with the ethical principles of the Declaration of Helsinki and the principles of Good Clinical Practice (ICH-GCP E6) [7]. This trial is registered at http://www.ClinicalTrials.gov (ID: NCT04055233).

### Patients and intervention

Patients 18 years or older, capable to give informed consent, and undergoing elective open or laparoscopically assisted abdominal surgery were eligible to participate. Patients were randomly assigned either to the control group with 0.9% saline or to the experimental group with 0.04% polyhexanide solution [6].

### Outcomes

In the RECIPE trial, SSI was defined according to the criteria by the Centers for Disease Control and Prevention within 30 days postoperatively (Table 1) [6]. Examination of the wound at least every second day, regularly dressing changes, and decision, if SSI was present, were performed on the surgical ward by attending surgeons. To evaluate SSIs after discharge, an interview by telephone was conducted at least 30 days postoperatively. Patients were asked whether the wound was closed or secondary healing and whether inpatient or outpatient medical treatment of the SSI was required. In case of uncertain wound sites, an appointment in our outpatient department was arranged for the patient. We classified SSI in superficial, deep, and organ/space SSI according to CDC (Table 1) [6]. An intraabdominal abscess was counted as organ/space SSI (grade 3) when it occurred in close proximity to the surgical incision.

**Table 1:** Definition of surgical site infection (SSI) according to Center for Disease Control [16]

Superficial incisional SSI	Infection occurs within 30 days after the operation AND Involves only skin and subcutaneous tissue of the incision AND patient has at least one of the following: 1. Purulent drainage from the superficial incision 2. Organisms isolated from an aseptically obtained culture of fluid or tissue from the superficial incision 3. At least one of the following symptoms: localized pain or tenderness; localized swelling; erythema; or heat and the superficial incision is deliberately opened by a surgeon unless incision is culture-negative 4. Diagnosis of superficial incisional SSI by the surgeon
Deep incisional SSI	Infection occurs within 30 days after the operation and the infection appears to be related to the operation AND Involves deep soft tissue of the incision (e.g., fascial and muscle layers) AND patient has at least one of the following: 1. Purulent drainage from the deep incision but not from the organ/space component of the surgical site 2. A deep incision spontaneously dehisces or is deliberately opened by a surgeon when the patient has at least one of the following symptoms: fever (> 38 °C); localized pain or tenderness; unless the site is culture-negative 3. An abscess or other evidence of infection involving the deep incision is found on direct examination, during reoperation, or by histopathologic or radiologic examination 4. Diagnosis of a deep incisional SSI by a surgeon
Organ/space SSI	Infection occurs within 30 days after the operation and the infection appears to be related to the operation AND Involves any part of the body deeper than fascial/muscle layers that were opened or manipulated during an operation AND patient has at least one of the following: 1. Purulent drainage from a drain that was placed into the organ/space 2. Organisms isolated from an aseptically obtained culture of fluid or tissue in the organ/space 3. An abscess or other evidence of infection involving the organ/space is found on direct examination, during reoperation or by histopathologic or radiologic examination 4. Diagnosis of an organ/space SSI by a surgeon

The endpoints of this post hoc analysis included the overall inpatient costs. The cost analysis referred to inpatient treatment. Data on costs were collected with the help of the tool of our corporate controlling team. The calculated costs of our study population only refer to the department for general and visceral surgery of Charité Berlin, Campus Benjamin Franklin, where the study was conducted. The calculation of costs is based on the official guidelines of the German Institute for remuneration in hospitals (InEK) for the calculation of diagnosis-related groups in Germany. The German Institute for remuneration in hospitals (InEK) sets these guidelines to ensure that the calculation of costs is comparable between all participating hospitals in Germany [8]. The tool of our corporate controlling team is a graphic display which outlines the calculation of costs and the comparison of proceeds and costs. This tool calculated the exact inpatient costs based on the case number of every single patient. These included overall costs of hospital treatment, surgery costs, costs on surgical ward, medication costs, laboratory costs, and costs of diagnostic procedures. Overall inpatient costs consisted of all categories that are depicted in Table 2. Surgery costs were mainly referring to cutting-suture time and time of anesthesia. Surgery costs also involved costs for revision surgery. Costs for medical material and drugs used in the operation room are included. In case of readmission within 30 days postoperatively, costs were added to the primary case number. In the case of reoperation surgery, costs of the second operation were added to the primary case number. Patients were interviewed by telephone at 30 days or greater postoperatively. Satisfaction with wound cosmesis was graded on a numeric analog scale (NAS) from 0–3 (not content) over 4–7 (moderately content) to 8–10 (very content). The parameter pain was assessed in the area of the scar or secondarily healing wound 30 days postoperatively. The pain was recorded on NAS ranging from 0 (no pain) to 10 (strongest pain imaginable).

**Table 2:**
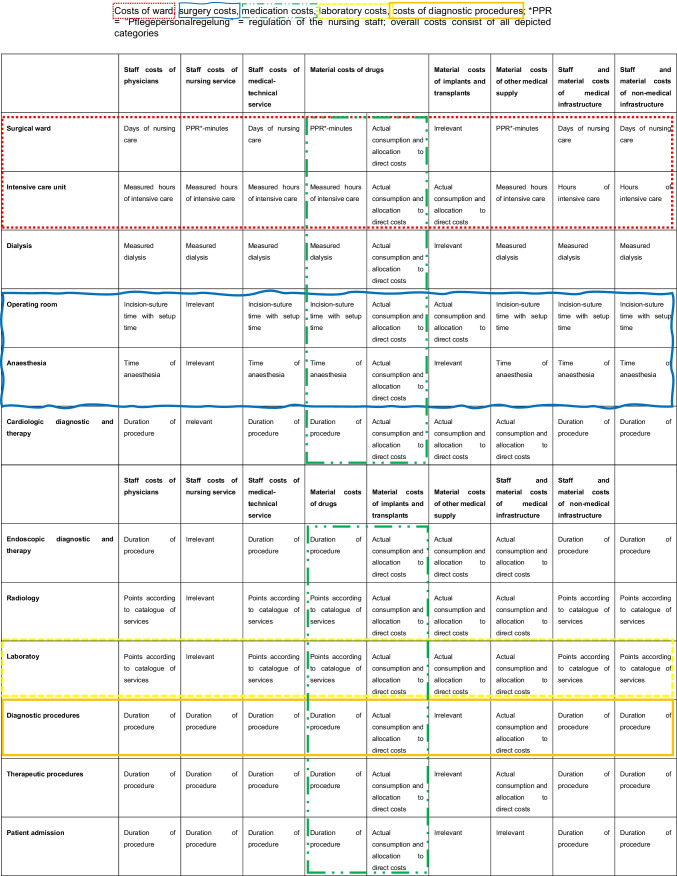
Composition of cost analysis

### Statistical analysis

Categorical variables of the primary outcome were analyzed with cross-tabulation and chi-square tests. For quantitative outcomes, statistical group comparisons were performed using the *t*-test for independent samples. Due to the skewed distribution of some of the quantitative variables, group differences were analyzed by Mann–Whitney *U* test or Kruskal–Wallis test. Additional parameters were depicted according to their scale and distribution with absolute and relative frequencies for categorical parameters and mean, standard deviation (SD), median, and interquartile range (IQR) for quantitative parameters; *p*-values ≤ 0.05 were considered as statistically significant. Statistical analysis was carried out using IBM SPSS Statistics 25® (IBM, Armonk, NY, USA).

## Results

### Patient characteristics

In the RECIPE trial, 456 patients were recruited and 393 (86.2%) completed the follow-up 30 days postoperatively [6]. Patients of this post hoc analysis had an average age of 58.3 ± 16.2 years. Overall, there were 154 (39.2%) female and 239 (60.8%) male patients. There was neither a difference in the duration of surgery nor in the organ site of the conducted operation between patients with and without SSI. Right- or left-sided hemicolectomy was performed in 23.9%, low anterior rectum resection in 13.8%, and colectomy or proctocolectomy in 13.0% (Table 3).

**Table 3:** Baseline and surgical characteristics

	SSI (*n* = 111)	No SSI (*n* = 282)	Total (*n* = 393)	*p*-value
Sex				0.91*
Female	43 (38.7%)	111 (39.4%)	154 (39.2%)	
Male	68 (61.3%)	171 (60.6%)	239 (60.8%)	
Age (years; mean ± SD)	56.6 ± 16.8	59.1 ± 15.9	58.3 ± 16.2	0.17┼
BMI (kg/m^2^, mean ± SD)	24.9 ± 4.7	25.3 ± 4.5	25.2 ± 4.6	0.48┼
Duration of surgery (min; mean ± SD)	253.3 ± 119.8	249.8 ± 123.0	250.8 ± 121.9	0.80┼
Organ site/type of operation				0.21*
Esophagus resection	4 (3.6%)	15 (5.3%)	19 (4.8%)	
Stomach resection	9 (8.1%)	16 (5.7%)	25 (6.4%)	
Liver resection	9 (8.1%)	29 (10.2%)	38 (9.7%)	
Pancreas resection	5 (4.5%)	18 (6.4%)	23 (5.8%)	
Small bowel resection	22 (19.8%)	27 (9.6%)	49 (12.5%)	
Ileocecal resection	4 (3.6%)	15 (5.3%)	19 (4.8%)	
Right- or left-sided hemicolectomy	26 (23.4%)	68 (24.1%)	94 (23.9%)	
Low anterior resection	15 (13.5%)	39 (13.9%)	54 (13.8%)	
Colectomy or proctocolectomy	14 (12.6%)	37 (13.1%)	51 (13.0%)	
Multivisceral resection	1 (0.9%)	5 (1.8%)	6 (1.5%)	
Others	2 (1.8%)	13 (4.6%)	15 (3.8%)	

Data are *n* (%) or mean ± SD; *SD*, standard deviation; *SSI*, surgical site infection; *BMI*, body mass index; *min*, minutes; *chi-square test; ┼*t*-test for independent samples

### Cost analysis

The overall inpatient costs for patients with SSI were 16.685 €; 19.703 USD median (IQR 21.638 €; 25.552 USD) compared to 11.235 €; 13.267 USD median (IQR 11.564 €; 13.656 USD) for patients without SSI (*p* < 0.001). Surgery costs differed in patients with SSI (6.664 €; 7.870 USD) to patients without SSI (5.040 €; 5.952 USD) (*p* = 0.001). Regarding costs on surgical ward, the occurrence of SSI was more expensive (8.404 €; 9.924 USD vs. 4.690 €; 5.538 USD; *p* < 0.001). The same refers to medication costs (663 €; 783 USD vs. 306 € (361 USD; *p* < 0.001) and laboratory costs (965 €; 1.140 USD vs. 592 €; 699 USD; *p* = 0.005) (Table 4). Additional costs directly caused by SSI were identified by excluding patients with severe postoperative complications (grades 3–5 according to Clavien–Dindo) in a supplementary analysis. Overall inpatient costs for patients with SSI without severe complication were 11.908 €; 14.038 USD median (IQR 4.933 €; 5.816 USD) vs. 9.354 €; 11.027 USD median (IQR 6.306 €; 7.434 USD) patients without SSI (*p* = 0.006). Costs on the surgical ward were 5.715 €; 6.737 USD median (IQR 4.568 €; 5.385 USD) for patients with SSI vs. 3.707 €; 4.370 USD median (IQR 2.988 €; 3.523 USD) for patients without SSI (*p* = 0.001).

**Table 4:** Cost analysis of SSI

	SSI (*n* = 103)	No SSI (*n* = 251)	Total (*n* = 354)	*p*-value*
Overall inpatient costs				< 0.001┼
Median	16.685 € (19.703 USD)	11.235 € (13.267 USD)	12.658 € (14.948 USD)	
IQR	21.638 € (25.552 USD)	11.564 € (13.656 USD)	14.140 € (16.742 USD)	
Q25	10.626 € (12.581 USD)	7.521 € (8.905 USD)	8.259 € (9.779 USD)	
Q75	32.264 € (38.200 USD)	19.085 € (22.597 USD)	22.399 € (26.520 USD)	
Surgery costs				0.001┼
Median	6.664 € (7.870 USD)	5.040 € (5.952 USD)	5.416 € (6.396 USD)	
IQR	6.550 € (7.735 USD)	3.992 € (4.714 USD)	4.865 € (5.760 USD)	
Q25	4.132 € (4.892 USD)	3.420 € (4.049 USD)	3.557 € (4.211 USD)	
Q75	10.682 € (12.647 USD)	7.412 € (8.776 USD)	8.423 € (9.973 USD)	
Costs on surgical ward				< 0.001┼
Median	8.404 € (9.924 USD)	4.690 € (5.538 USD)	5.533 € (6.534 USD)	
IQR	15.099 € (17.830 USD)	6.456 € (7.624 USD)	8.099 € (9.589 USD)	
Q25	4.430 € (5.245 USD)	3.067 € (3.631 USD)	3.305 € (3.913 USD)	
Costs on surgical ward				
Q75	19.529 € (23.122 USD)	9.523 € (11.275 USD)	11.403 € (13.501 USD)	
Medication costs				< 0.001┼
Median	663 € (783 USD)	306 € (361 USD)	371 € (438 USD)	
IQR	1.798 € (2.123 USD)	663€ (783 USD)	1.152 € (1.364 USD)	
Q25	273 € (323 USD)	179 € (212 USD)	191 € (226 USD)	
Q75	2.071 € (2.452 USD)	842 € (997 USD)	1.344 € (1.591 USD)	
Laboratory costs				0.005┼
Median	965 € (1.140 USD)	592 € (699 USD)	661 € (781 USD)	
IQR	1.742 € (2.057 USD)	988 € (1.167 USD)	1.079 € (1.278 USD)	
Q25	330 € (391 USD)	286 € (339 USD)	296 € (350 USD)	
Q75	2.072 € (2.453 USD)	1.275 € (1.510 USD)	1.375 € (1.628 USD)	
Costs of diagnostic procedures				0.13
Median	60 € (71 USD)	33 € (39 USD)	40 € (47 USD)	
IQR	242 € (286 USD)	103 € (122 USD)	143 € (169 USD)	
Costs of diagnostic procedures				
Q25	9 € (11 USD)	9 € (11 USD)	9 € (11 USD)	
Q75	251 € (297 USD)	111 € (131 USD)	152 € (180 USD)	

Data are € and USD; *IQR*, interquartile range; *Q*25, 0.25 quartile; *Q*75, 0.75 quartiles; exchange rate from € in USD based on 11/12/2020; *SSI*, surgical site infection; *Mann–Whitney *U* test; ┼*p* ≤ 0.05

Overall inpatient costs for patients who were irrigated with saline in the RECIPE trial were 12.056 €; 14.237 USD median (IQR 13.612 €; 16.074 USD) vs. 12.793 €; 15.107 USD median (IQR 12.085 €; 14.271 USD) in the polyhexanide group (*p* = 0.52). Surgery costs amounted to 5.156 €; 6.089 USD) in the saline and 5.596 € (6.608 USD) in the polyhexanide group (*p* = 0.53).

Surgery costs varied depending on the performed procedure. For example, costs for esophagus resection were 11.586 €; 13.682 USD median, for pancreas resection 8.473 €; 10.006 USD median, and for right- or left-sided hemicolectomy 4.055 €; 4.789 USD median; *p* < 0.001 (Table 5).

**Table 5:** Surgery costs depending on the procedure

	Surgery costs (*n* = 392)	*p*-value*
Surgical procedure		<0.001┼
Esophagus resection	11.586 € (13.682 USD)	
Stomach resection	7.098 € (8.382 USD)	
Major liver resection	6.582 € (7.773 USD)	
Minor liver resection	4.978 € (5.879 USD)	
Pancreas resection	8.473 € (10.006 USD)	
Small bowel resection	3.787 € (4.472 USD)	
Ileocecal resection	3.379 € (3.990 USD)	
Right- or left-sided hemicolectomy	4.055 € (4.789 USD)	
Low anterior resection	5.641 € (6.661 USD)	
Abdominoperineal excision	9.459 € (11.170 USD)	
Colectomy, proctocolectomy, proctectomy, and ileoanal pouch	6.646 € (7.848 USD)	
Multivisceral resection and others	3.547 € (4.189 USD)	

Data are median, € and USD; exchange rate from € in USD based on 11/12/2020; *Kruskal–Wallis test; ┼*p* ≤ 0.05

### Postoperative outcome

Overall, we recorded 111 (28.2%) SSIs. Sixty-six (59.5%) of SSI were detected during inpatient treatment and 45 (40.5%) occurred within 30 days postoperatively. Data about the satisfaction with the cosmetic result 30 days postoperatively were documented in 318 patients. Eighteen patients with SSI (16.2%) and twelve patients without SSI (4.3%) were not content (*p* < 0.001) (Table 6).

**Table 6:** Impact of SSI on postoperative outcome

	SSI (*n* = 111)	No SSI (*n* = 282)	Total (*n* = 393)	*p*-value
Satisfaction with cosmetic result				< 0.001*§
Not content (0–3)	18 (16.2%)	12 (4.3%)	30 (7.6%)	
Moderately content (4–7)	36 (32.4%)	84 (29.8%)	120 (30.5%)	
Very content (8–10)	20 (18.0%)	148 (52.5%)	168 (42.7%)	
Missing data	37 (33.3%)	38 (13.5%)	75 (19.1%)	
Medical treatment of SSI after discharge	75 (67.6%)	0 (0%)	75 (19.1%)	< 0.001*§
Inpatient treatment after discharge	6 (5.4%)	0 (0%)	6 (1.5%)	< 0.001*§
Outpatient treatment after discharge	69 (62.2%)	0 (0%)	69 (17.6%)	< 0.001*§
Missing data	6 (5.4%)	9 (3.2%)	15 (3.8%)	
Postoperative pain on NAS (mean ± SD)	1.3 ± 1.7	0.6 ± 1.4	0.8 ± 1.5	< 0.001*‡
Postoperative pain on NAS				< 0.001*§
None (0)	46 (41.4%)	201 (71.3%)	247 (62.8%)	
Mild (1–3)	48 (43.2%)	64 (22.7%)	112 (28.5%)	
Moderate to strong (4–10)	4 (3.6%)	4 (1.4%)	8 (2.0%)	
Missing data	13 (11.7%)	13 (4.6%)	26 (6.6%)	
Total length of hospital stay (days; median)	18.0	12.0	14.0	< 0.001*┼
Severe postoperative complication (Clavien–Dindo grades 3–5) until discharge	48 (43.2%)	67 (23.8%)	115 (29.3%)	< 0.001*§
Reoperation in general anesthesia total	52 (46.8%)	59 (20.9%)	111 (28.2%)	< 0.001*§
Reoperation due to SSI and fascia dehiscence	20 (18.0%)	11 (3.9%)	9 (2.3%)	
Reoperation not due to SSI	32 (28.8%)	48 (17.0%)	102 (25.9%)	

Data are *n* (%) or median or mean ± SD; *SSI*, surgical site infection; *p ≤ 0.05; **┼**Mann–Whitney *U* test; *NAS*, numerical analogous scale; *SD*, standard deviation; ‡*t*-test for independent samples; §chi-square test; the number of patients differs between the categories because follow-up was not accomplished by all patients

Seventy-five (67.6%) patients with SSI required medical treatment of SSI again after discharge. Inpatient medical treatment after discharge was necessary in six (5.4%) patients and outpatient treatment in 69 (62.2%) patients with SSI (*p* < 0.001).

The total length of hospital stay including readmission was 18 days median (patients with SSI) compared to 12 days median (patients without SSI) (*p* < 0.001) [6]. In the group with SSI, postoperative pain at the scar 30 days postoperatively was rated 1.3 ± 1.7 SD on average on NAS compared to 0.6 ± 1.4 SD in the group without SSI (*p* < 0.001) (6). Patients without SSI complained less often about postoperative pain at the scar than patients with SSI: 71.3% vs. 41.4% had no pain (*p* < 0.001) [6].

## Discussion

SSI is one of the most frequent complications in visceral surgery. Still, data on the economic impact of SSI on the healthcare system in Germany and on patients’ quality of life is rare. To our knowledge, this analysis is the first detailed economic evaluation of inpatient healthcare costs based on the tool of the corporate controlling team of the surgical department of Charité – Universitätsmedizin Berlin. Overall inpatient costs were higher in patients with SSI after elective laparotomy. SSI led to more postoperative pain and less satisfaction with the cosmetic result of the operation 30 days postoperatively.

This post hoc analysis was an integral part of the monocentric randomized controlled RECIPE trial with 456 patients that investigated the benefits of antiseptic intraoperative wound irrigation on SSI in visceral surgery. Healthcare costs were evaluated using the tool of our corporate controlling team based on the G-DRG system. This tool calculates the exact costs based on the case number of every single patient which provides accurate cost estimates to understand the true burden of SSI. In case of readmission within 30 days postoperatively, the costs were added to the primary case number.

The overall median inpatient costs for patients with SSI accounted for 16.685 € (19.703 USD) and were therefore higher than for patients without SSI (11.235 €; 13.267 USD). Regarding the subcategories surgery costs, costs of the surgical ward, medication, and laboratory costs, all of these were more expensive in the group with SSI. As stated previously in the RECIPE trial, the length of hospital stay was longer in patients with SSI [6]. This is one factor possibly increasing costs on a surgical ward for patients with SSI and costs for medication and laboratory. One factor that might have influenced the higher costs of patients with SSI in this analysis is the concomitant incidence of severe postoperative complications (grades 3–5 according to Clavien–Dindo). Nearly 29% of patients with SSI required reoperation in general anesthesia, not due to SSI. A comparison of patients with SSI but no further complication and patients without SSI revealed that overall inpatient costs were approximately 2.500 € (3.000 USD) higher when SSI occurred. Eighty percent of the increase in costs was caused by costs on a surgical ward.

Previous trials also presented increasing healthcare costs due to SSI. In a single-center, case matched follow-up study, Kirkland et al. [9] showed that the median direct costs of hospitalization were 7.531 USD for patients with SSI and 3.844 USD for patients without SSI. The mean additional cost per patient with SSI was £10.523 (13.864 USD) calculated from actual resources used by each patient in a British surveillance program in colorectal surgery [10]. Economic data in our study were collected in a prospective randomized controlled setting. The cost assessment tool was not based on any economic model such as in some of the aforementioned studies. Our tool provides accurate data based on every patient’s individual hospital case.

In this analysis, the impact of SSI on the 30-day morbidity of patients was clearly shown. The total length of hospital stay was 6 days longer in the median when SSI occurred during inpatient treatment, as described previously in the RECIPE trial [6]. Prolongation of hospital stay because of SSI was also seen in a single-center, case-matched follow-up study by Kirkland et al. [9]. A systematic review of six European countries outlined that SSI was associated with elevated healthcare costs and prolonged hospitalization [11]. Pinkney et al. [12] reported an 11% reduction in health-related quality of life 30 days postoperatively in patients with SSI after laparotomy. A systematic review by Gheorghe et al. [13] again identified a lack of studies that investigate the impact of SSI particularly in abdominal surgery on quality of life.

To our knowledge, this is the first study to evaluate the impact of SSI on subjective parameters such as pain and satisfaction with the cosmetic result. Patients with SSI complained more often about pain at the scar 30 days postoperatively than patients without SSI. They rated higher on the 10-point numeric analogous scale for pain. Additionally, patients who developed SSI were less content with the cosmetic result of the scar 30 days postoperatively. It must be recognized that postoperative pain and satisfaction with the cosmetic result are two subjective parameters.

The overall detected SSI rate of 28% in elective visceral surgery is above the average SSI rate of other trials. The German National Reference Center for the surveillance of nosocomial infections depicts nearly 11% of SSI following colorectal surgery referring to patients during the period of hospitalization [14]. The comprehensive detection of SSI by thorough and regular examination on the ward and the exact capturing of outpatient SSI are two main reasons for the high rate of SSI in our study. Data concerning only hospitalized patients might not be representative. Other trials focusing on SSI as a primary endpoint found similarly high rates of SSI. The overall rate of SSI was 21.4% in a German multicenter randomized controlled trial focusing on circular plastic wound edge protectors in laparotomy [15]. A British multicenter randomized trial addressing the impact of wound edge protection devices on SSI reported a similarly high rate of 25% SSI [12].

In the RECIPE trial, evidence is provided that intraoperative subcutaneous wound irrigation with antiseptic polyhexanide solution reduces SSI in elective laparotomies compared to saline [6]. One research question was whether median inpatient costs were cheaper in the group irrigated with polyhexanide. The price of 250 ml of antiseptic 0.04% polyhexanide solution (Serasept2®) is currently 4.93 € (5.52 USD) in Germany in an inpatient setting [6]. The price of 250 ml of 0.9% saline is currently 1.05 € (1.14 USD) in Germany. There was no difference in median overall inpatient costs between the two irrigation solutions. Total inpatient costs were probably more influenced by even more severe postoperative complications or patients’ comorbidities, showing the cost effect of the irrigation solution.

Several potential limitations of the trial must be taken into account. First, this was a monocenter randomized trial with inherent limitations. Perioperative settings, operative procedures, and patient characteristics may vary from the ones in other nonacademic German or international institutions. The calculated costs of our study population only refer to the department for general and visceral surgery of Charité Berlin, Campus Benjamin Franklin, where the study was conducted. Second, it should be noted that costs caused by SSI in the outpatient sector are not depicted by this trial. Nearly 41% of SSI in this trial occurred after discharge. Wounds healing by secondary intent require outpatient wound care with regularly dressing changes which additionally increase healthcare costs for patients with SSI. So it is possible that the true economic burden of SSI is underestimated. Tanner et al. [10] showed in a post-discharge surveillance program of SSI that costs for wound care after discharge accounted for 15% of the total costs. Third, there was no clinical examination 30 days postoperatively, leaving the chance of missing some SSI or capturing some delayed wound healings as SSI which did not fulfill the CDC criteria. We strictly complied with the follow-up time of 30 days postoperatively and interviewed the patients by telephone [6]. Fourth, a variety of surgical procedures was included in the RECIPE trial as all elective laparotomies or laparoscopically assisted surgeries were eligible for inclusion. There was no difference in the rate of SSI depending on the organ site. A trend towards more SSI in small bowel resection was shown. The subgroups of surgical procedures might be underpowered, and therefore, no difference in the rate of SSI was found in our trial.

Taken these limitations into account, this post hoc analysis of a large-scale prospective randomized trial with comprehensive cost analysis showed a marked increase in inpatient costs for patients with SSI. Furthermore, patients with SSI reported more pain and worse cosmetic results in postoperative 30-day follow-up.

## Conclusion

This post hoc analysis of the randomized controlled RECIPE trial provided evidence that SSI has a major impact on patients’ postoperative course. Inpatient healthcare costs due to SSI are increased. Therefore, the prevention of SSI is crucial. Overall costs for intraoperative wound irrigation with saline were comparable with polyhexanide.

## Data Availability

This trial is registered at http://www.ClinicalTrials.gov (ID: NCT04055233).
